# BCG-Unresponsive Non-Muscle-Invasive Bladder Cancer: Current Treatment Landscape and Novel Emerging Molecular Targets

**DOI:** 10.3390/ijms241612596

**Published:** 2023-08-09

**Authors:** Francesco Claps, Nicola Pavan, Luca Ongaro, Domenico Tierno, Gabriele Grassi, Carlo Trombetta, Gabriele Tulone, Alchiede Simonato, Riccardo Bartoletti, Laura S. Mertens, Bas W. G. van Rhijn, Maria Carmen Mir, Bruna Scaggiante

**Affiliations:** 1 Urological Clinic, Department of Medicine, Surgery and Health Sciences, University of Trieste, 34149 Trieste, Italy; claps.francesco@gmail.com (F.C.); ongarluc@gmail.com (L.O.); trombcar@units.it (C.T.); 2 Department of Surgical Oncology (Urology), Netherlands Cancer Institute, Antoni van Leeuwenhoek Hospital, 1066 CX Amsterdam, The Netherlands; ls.mertens@gmail.com (L.S.M.); basvanrhijn@hotmail.com (B.W.G.v.R.); 3 Department of Surgical, Oncological, and Oral Sciences, University of Palermo, 90127 Palermo, Italy; nicpavan@gmail.com (N.P.); gabriele.tulone@gmail.com (G.T.); alchiede.simonato@unipa.it (A.S.); 4 Department of Life Sciences, University of Trieste, 34127 Trieste, Italy; tiernodomenico@gmail.com; 5 Department of Medical, Surgery and Health Sciences, Hospital of Cattinara, University of Trieste, Strada di Fiume 447, 34149 Trieste, Italy; ggrassi@units.it; 6 Department of Translational Research and New Technologies, University of Pisa, 56126 Pisa, Italy; riccardo.bartoletti@unipi.it; 7 Department of Urology, Hospital Universitario La Ribera, 46600 Valencia, Spain; mirmare@yahoo.es

**Keywords:** non-muscle-invasive bladder cancer, Bacillus Calmette–Guérin-unresponsive non-muscle-invasive bladder cancer, bladder sparing treatment, chemo-hyperthermia, immunotherapy, gene therapy, photodynamic therapy

## Abstract

Urothelial carcinoma (UC), the sixth most common cancer in Western countries, includes upper tract urothelial carcinoma (UTUC) and bladder carcinoma (BC) as the most common cancers among UCs (90–95%). BC is the most common cancer and can be a highly heterogeneous disease, including both non-muscle-invasive (NMIBC) and muscle-invasive (MIBC) forms with different oncologic outcomes. Approximately 80% of new BC diagnoses are classified as NMIBC after the initial transurethral resection of the bladder tumor (TURBt). In this setting, intravesical instillation of Bacillus Calmette–Guerin (BCG) is the current standard treatment for intermediate- and high-risk patients. Unfortunately, recurrence occurs in 30% to 40% of patients despite adequate BCG treatment. Radical cystectomy (RC) is currently considered the standard treatment for NMIBC that does not respond to BCG. However, RC is a complex surgical procedure with a recognized high perioperative morbidity that is dependent on the patient, disease behaviors, and surgical factors and is associated with a significant impact on quality of life. Therefore, there is an unmet clinical need for alternative bladder-preserving treatments for patients who desire a bladder-sparing approach or are too frail for major surgery. In this review, we aim to present the strategies in BCG-unresponsive NMIBC, focusing on novel molecular therapeutic targets.

## 1. Introduction

Urothelial carcinoma (UC), which includes bladder carcinoma (BC) and upper urinary tract urothelial carcinoma (UTUC), is the sixth most common cancer in Western countries [1]. BC, as the most common cancer, is a highly heterogeneous disease that includes both non-muscle-invasive bladder carcinoma (NMIBC) and muscle-invasive bladder carcinoma (MIBC) with different oncologic outcomes [2,3,4]. Approximately 80% of newly diagnosed bladder carcinomas are classified as NMIBC after the initial transurethral resection of the bladder tumor (TURBt), representing a broad spectrum of disease [5]. While low-risk NMIBC patients are mainly treated with TURBt alone, intermediate- and high-risk patients often receive adjuvant treatments to reduce disease recurrence and progression [5,6,7]. In this context, intravesical administration of Bacillus Calmette–Guerin (BCG) is the current standard treatment [8]. Despite adequate BCG treatment, recurrence occurs in approximately 40% of patients and MIBC in 15% [9]. In particular, highly recurrent BCs represent the highest risk of disease [8]. The European Association of Urology guidelines (EAU) currently define “BCG-unresponsive” as all BCG-refractory tumors and those that develop T1/Ta HG recurrence within six months of completion of adequate BCG exposure or develop carcinoma in situ (CIS) within twelve months of completion of adequate BCG exposure. “Adequate BCG exposure” is defined as the completion of at least five of six doses of a first induction course plus at least two of six doses of a second induction course or two of three doses of a maintenance regimen. Patients with NMIBC who do not respond to BCG are extremely unlikely to benefit from further BCG administration and represent a patient cohort for whom new treatment options are urgently needed [10]. High-risk NMIBCs who do not respond to BCG, therefore, present a therapeutic challenge.

In this specific scenario, non-surgical options are limited, and according to the EAU guidelines [5,11], the recommended treatment for BCG-unresponsive disease remains radical cystectomy (RC) and urinary diversion (UD). RC and UD remain complex major urological procedures with a recognized high perioperative morbidity due to patient, disease, and surgical factors [12,13]. Numerous improvements have been made in surgical technique and perioperative management [14]. However, the morbidity profile and survival after RC have remained largely unchanged [15,16,17]. As this is a predominantly elderly disease, a non-negligible number of patients are considered unsuitable for such surgery [16].

Despite the unsatisfactory efficacy profile, with a complete response rate (CR) of about 18%, by 2020 the only Food and Drug Administration (FDA)-approved conservative treatment for CIS patients who did not respond to BCG was intravesical valrubicin [18]. Nowadays, new therapeutic options, potentially ready for the first time, could lead to a turning point in the treatment of patients who do not respond to BCG. In such a scenario, two unmet clinical needs could emerge. First, reliable biomarkers could identify early those patients who do not respond to BCG immunotherapy. Second, the current arsenal of new intravesical and systemic treatment candidate agents could be expanded for bladder-sparing strategies. If the identification of new molecular biomarkers is of interest for all disease stages of BC [19,20,21], the choice of new treatment candidates for NMIBC is wide, leading the FDA to accept single-arm clinical trials as adequate clinical evidence for evaluating therapeutics [22,23,24]. The choice of new treatment candidates for NMIBC is wide, leading the FDA to accept single-arm clinical trials as adequate clinical evidence for evaluating therapeutics.

In this review, strategies for BCG-unresponsive NMIBC are presented with the aim of examining the role of alternative therapies for NMIBC, focusing on the new molecular targets.

## 2. Intravesical Chemotherapy

**Figure 1:**
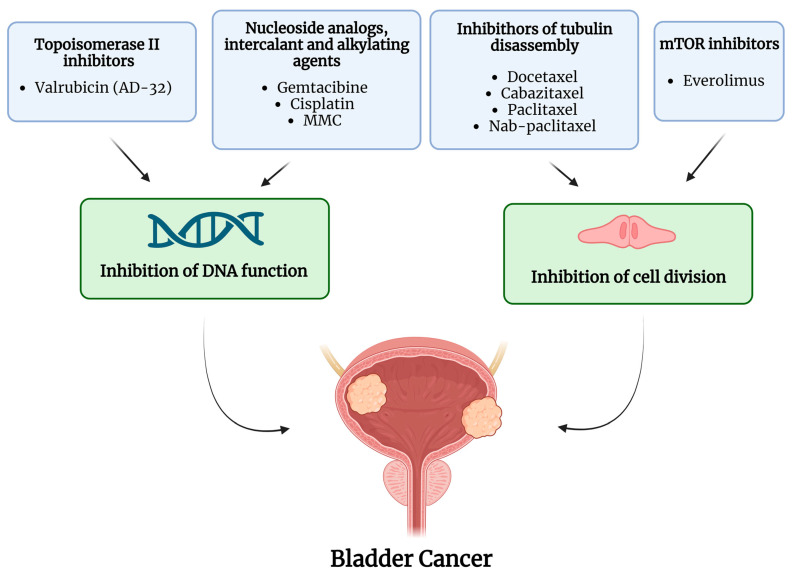
Active molecules and mechanism of action of intravesical chemotherapy. The image was created with BioRender.com.

Over the years, intravesical administration of various chemotherapeutic agents has been investigated in BCG-unresponsive NMIBC. Figure 1 summarizes the drugs and mechanisms of action of intravesical chemotherapies; Table 1 summarizes the studies on intravesical treatments and the main findings over the last twenty years.

In this scenario, among the chemotherapeutic agents studied, gemcitabine, a deoxycytidine nucleoside analog that can inhibit DNA synthesis and is a cornerstone in the systemic treatment of MIBC in both neoadjuvant and adjuvant settings, may be the one that receives more attention [24]. Gemcitabine has been administered as a single agent by some authors [25,37,38,39,40,41,42,43,44,45,46,47,48,49,51,52,53], while other series have tested combinations with various other drugs such as docetaxel [25,27,29,33], cabazitaxel and cisplatin [28], oral everolimus [30], and mitomycin C (MMC) [32,34]. Gemcitabine administration regimens are quite heterogeneous in the published literature, with some studies suggesting an induction regimen only [38,47,48,51,53] and others recommending a maintenance regimen in responders [26,37,42,43,45,52]: no clear and standardized regimen can be found in either single-agent or drug combination studies.

Regarding the oncologic outcomes of intravesical gemcitabine, there is considerable heterogeneity in the assessment of its efficacy and results. In addition, there is a non-negligible discrepancy in study designs, some of which are retrospective. For example, in 2020, Hurle et al. found disease-free survival (DFS) at 12 and 24 months of 44.44% and 31.66%, respectively, in their open-label, single-arm study in which gemcitabine alone was administered to 36 patients in an induction regimen followed by maintenance therapy in responders. In this study, progression-free survival (PFS) at 12 and 24 months was 80.13% and 69.55%, respectively, while cancer-specific survival (CSS) and overall survival (OS) evaluated at 24 months were 80.68% and 77.9%, respectively [26]. Skinner et al. tested gemcitabine alone in a phase II trial enrolling 58 patients and achieved a CR, i.e., negative cystoscopy, negative urine cytology, and negative biopsy, in 47% of patients at 3 months. The median recurrence-free survival (RFS) was 6.1 months and was still 21% at 24 months, with a progression rate of 36% to RC. In this study, an induction regimen was used, followed by maintenance therapy in responders [38]. According to the retrospective data of Sternberg et al. on 69 patients treated with gemcitabine induction alone, the 5-year progression to MIBC rate was 19% in BCG-unresponsive patients and 22% in patients with other types of BCG failure. In this series, 27 patients achieved a CR (negative cystoscopy and urinary cytology), 19 achieved a partial response (negative cystoscopy and positive cytology), and 20 patients experienced failure (positive cystoscopy). The cancer-specific death rate was 12% in the complete responders and 18% in the remainder. Subsequent RC had to be performed on 20 patients [38]. Combinations of gemcitabine with other drugs produced different outcomes. According to Chevuru and colleagues, gemcitabine plus docetaxel resulted in an RFS at 12, 24, and 60 months of 57%, 44%, and 24%, respectively, and an HG-RFS at 12, 24, and 60 months of 60%; remarkably, outcomes were slightly worse only in CIS patients compared to papillary disease-only ones [25]. Similar results were found in the retrospective study by Steinberg et al. published in 2020 [27], while the same drug combination performed worse in the study by Milbar [29]. De Castro reported encouraging results by combining gemcitabine with cabazitaxel and cisplatin: The triplet achieved a CR in 89% of cases with a median RFS of 27 months. RC-free survival was 94% at 1 year and 81% at 2 years [28]. The combination with oral Everolimus proposed by Dalbagni resulted in an RFS of 58% at 3 months, 27% at 6 and 9 months, and 20% at 12 months [30]. The association with MMC was tested by Cockerill and Lightfoot. Cockerill determined a recurrence rate of 63% with a median RFS of 15.2 months and a recurrence-free rate of 37% with a final median of 22 months after treatment [32]. Lightfoot found a CR rate and 1-year and 2-year RFS of 68%, 48%, and 38%, respectively [34].

Other drugs such as paclitaxel, nab-paclitaxel [31,40,41], valrubicin, and doxorubicin [33,46] have been studied with conflicting results. Docetaxel alone resulted in a CR in 56% to 59% of patients [36,40]. In summary, despite some promising results or at least some effect in certain cases, no definitive conclusion can be drawn regarding intravesical chemotherapy after BCG administration because of the heterogeneity of the studies and protocols and the limited number of patients enrolled in most of these studies.

## 3. Chemo-Hyperthermia

**Figure 2:**
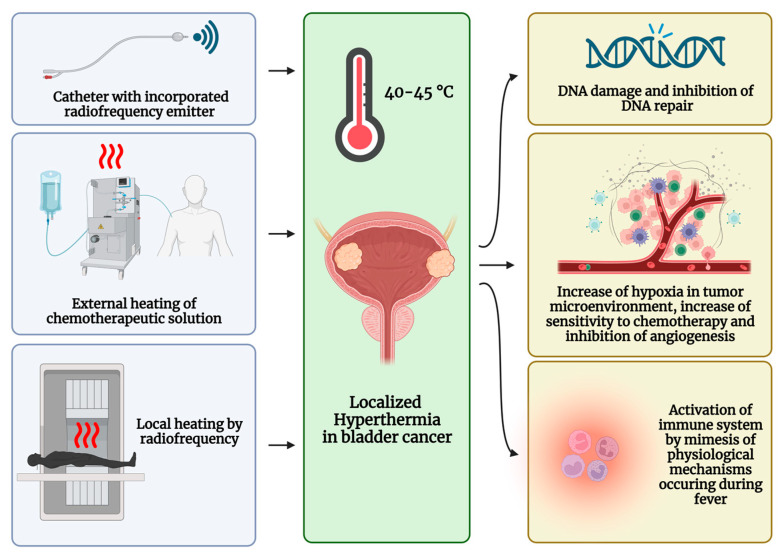
Scheme and mechanism of chemo—hyperthermia treatment. The image was created with BioRender.com.

The effect of hyperthermia on tumor cells has been known for decades, and several effects of hyperthermia have been described to date. First, at temperatures above 40.5 °C and above, ribonucleic acid (RNA) and deoxyribonucleic acid (DNA) synthesis is reduced, and DNA repair itself is impaired [54]. In addition, hyperthermia can alter tumor blood flow in several ways. Up to a temperature of 43 °C, vasodilatation is observed, which allows better delivery of chemotherapeutic agents. Moreover, direct damage such as occlusion and destruction of the endothelial cells lining the tumor vessels, which have a reduced ability to dissipate heat compared to normal tissue, has been described, resulting in decreased blood flow to the cancer cells [55]. In turn, the cancer microenvironment becomes increasingly hypoxic, and cancer cells become more sensitive to both heat and chemotherapy, while angiogenesis is inhibited via the upregulation of the plasminogen activator inhibitor-1 pathway in endothelial cells of the tumor vasculature [55]. In addition, hyperthermia mimics the physiological mechanisms occurring in the immune system during fever, such as the activation of CD8 and CD4 immune cells, the infiltration of NK cells, and the production of heat shock proteins and cytokines, thus facilitating the activation of many molecular effectors of the immune system [56]. In practice, an intravesical temperature ranging between 40 °C and 45 °C can be achieved either by using microwaves generated by a radiofrequency emitter incorporated in a catheter or by using a conductive system that externally heats the chemotherapeutic solution before intravesical administration. A further option is locoregional hyperthermia, which entails using an external device to generate local heating by radiofrequency [57]. A scheme of the hyperthermia treatment is shown in Figure 2.

Previous studies focused on the synergistic effect of hyperthermia in combination with MMC, an alkylating agent administered intravesical that is more effective at high temperatures [58]. Table 2 summarizes the results of studies investigating chemo-hyperthermia with MMC.

Despite some discrepancies between different experiences in terms of the treatment schedule and study design, DFS reached peaks of 85% in some series [65], with most of them reporting a CR in more or less 50% of patients at 1 or even 2 years [60,64,65]. Remarkably, the available data show that chemo-hyperthermia performed worse in patients with CIS [60] and in patients with papillary disease and concomitant CIS [61]. In a randomized clinical trial by Tan and colleagues, a comparison between radiofrequency-induced thermo-chemotherapy with MMC and BCG retreatment was made, with no statistically significant differences in oncological outcomes between the two arms [61].

In conclusion, despite some interesting results, the data need to be confirmed by further studies before chemo-hyperthermia can be incorporated into routine clinical practice.

## 4. Immunotherapy and Inflammation-Targeted Agents

**Figure 3:**
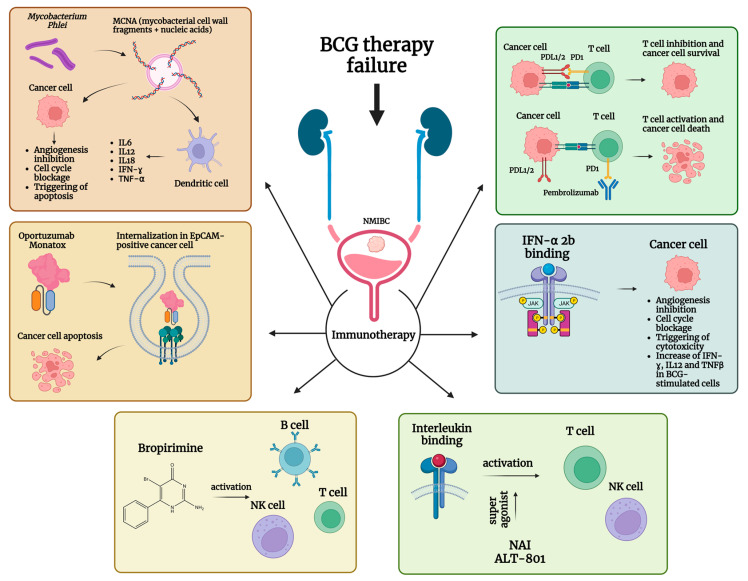
Schematic summary of immunotherapeutic approaches for patients with upper non-muscle-invasive bladder carcinoma (NMIBC) unresponsive to treatment with Bacillus Calmette–Guerin (BCG). Abbreviations are as follows: EpCAM: epithelial cell adhesion molecule; IFN: interferon; IL: interleukin; MCNA: mycobacterium phlei cell wall-nucleic acid complex; NAI: nogapendekin alfa inbakicept; NK cell: natural kill cell; PD-1: programmed cell death protein 1; PD-L1/2: programmed cell death protein ligand 1; TNF: tumor necrosis factor. The image was created with BioRender.com.

In the field of immunotherapy for both MIBC and UTUC, pembrolizumab has played an increasingly important role in recent years [11,66]. Pembrolizumab is a humanized monoclonal antibody against programmed cell death protein 1 (PD-1), a so-called immune checkpoint inhibitor (ICI). PD-1 is a transmembrane receptor that binds to two different ligands: Programmed cell death protein 1 ligand 1 (PD-L1) and ligand 2 (PD-L2). PD-1 is expressed by activated CD4C and CD8C T cells, B cells, natural killer (NK) cells, macrophages, and dendritic cells, whereas PD-L1 is constitutively expressed on T cells, B cells, dendritic cells, regulatory T cells (Treg), monocytes, and macrophages. Binding of PD-1 to its ligands PD-L1 and 2 results in an inhibitory transduction signal for T cells. Taking advantage of this mechanism, tumor cells express PD-L1 and thus evade the immune response. By binding PD-1 and preventing interaction with its ligands, pembrolizumab may enhance the anti-tumor immune response [67]. In light of this, some studies have attempted to evaluate the efficacy of pembrolizumab in BCG-refractory NMIBC. In such reports, pembrolizumab has been administered in combination with BCG by both intravesical [68] and intravenous routes and as a single agent [67]. In a series of 9 patients treated with a combination of BCG and intravesical pembrolizumab, Meghani et al. reported an RFS at 3, 6, and 12 months of 100%, 67%, and 22%, respectively. In this series, PFS at 6 and 12 months was 100% and 56%, respectively, with MIBC occurrence being reported in 5 patients [69]. Alanee et al. instead found a CR rate of 69% in 18 patients with BCG in combination with intravenous pembrolizumab. In this study, one patient progressed to MIBC [67]. In a series of 96 patients, intravenous pembrolizumab alone resulted in a CR in 39 cases with a 51% recurrence rate, and PFS to MIBC or metastatic disease or death was 97% [10]. Pembrolizumab was recently approved by the FDA for the treatment of BCG-unresponsive NMIBC. Although a phase II trial achieved CR in 41% of patients treated with pembrolizumab, less than 50% of responders continued to receive maintenance therapy [10]. Table 3 lists the studies of immunotherapy for BCG-unresponsive NMIBC. A schematic summary of the immunotherapeutic approaches described is reported in Figure 3.

Other strategies, such as the combination of BCG with interferon (IFN)α2b, have been tested. IFNα2b is a glycoprotein produced by different cell types in response to exposure to various foreign antigens, including tumor antigens. This protein exhibits a wide range of direct and indirect anti-tumoral effects. Direct effects include inhibition of angiogenesis, cytotoxicity, and cell cycle blockage [80,81]. Indirect effects include increased production of IFN-ɣ, interleukin (IL) 12, and tumor necrosis factor (TNF)-β in BCG-stimulated cells, which may shift the polarization of the immune system toward a T-helper (Th) 1 cytotoxic response and enhance the effects of BGC itself. Despite some in vitro results, it must be acknowledged that a clear advantage over BCG alone in terms of recurrence and progression rates to date has not yet been demonstrated [82]. However, DFS ranging from 42% to 53% at 24 months and up to 55% at 30 months [75,76] have been reported.

Other studies investigated the role of mycobacterium phlei cell wall-nucleic acid complex (MCNA), an immunomodulatory and antineoplastic compound derived from mycobacterium phlei that contains mycobacterial cell wall fragments complexed with biologically active nuclear acids. MCNA induces the synthesis of IL-6, IL-8, IL-12, IL-18, and TNF-α, which lead to an anticancer immune response by promoting the development of Th1 lymphocytes, stimulating the production of IFN-ɣ, inhibiting angiogenesis, and enhancing the activity of NK and cytotoxic T cells. In addition, MCNA can selectively induce apoptosis in cancer cells via translocation of phosphatidyl serine at the cell surface, nucleosome DNA fragmentation, release of soluble protein fragments of the mitotic nuclear apparatus, and cytochrome c from mitochondria [83]. A CR rate with MCNA was observed in up to 34.4% of patients at 6 months follow-up, with response rates decreasing thereafter [72,74]. Of note, MCNA appeared to perform worse in CIS patients.

In 2012, Kowalski et al. tested the efficacy of oportuzumab monatox (OM), an anti-epithelial cell adhesion molecule (EpCAM) single-chain humanized antibody linked to Pseudomonas exotoxin A. OM binds to EpCAM and is internalized into the cancer cell cytosol, where the toxin induces apoptosis. The authors reported a CR rate of 44%, which proved durable in 16% of the patients at 1 year. The 1-year recurrence rate was 65% [73].

Bromopirimine, an oral immunomodulatory agent, was also explored in the late 1990s. Bromopirimine displays several effects on the immune system, such as stimulating the proliferation of B cells and thus increasing serum immunoglobulin levels, enhancing macrophage-mediated and NK cell cytotoxicity, and activating T cells [84]. Overall, a CR of 24% was found with an RFS ranging from 65 to 810 days in patients [79]. In short, despite some interesting perspectives, clear-cut evidence for immunotherapy in BCG refractory NMIBC is still lacking.

The relationship between inflammation and tumor development has always been an important field of translational research [85]. Moreover, targeting inflammation in cancer treatment, including cyclooxygenase (COX), nuclear factor-kB (NF-kB), cytokines, and/or chemokines and their receptors, represents a crucial point in tumor invasion and progression, promoting the pathway as an integral part of enhanced angiogenesis [86]. Impaired standard laboratory inflammatory biomarkers have been associated with advanced disease and worse oncological outcomes [87]. In particular, non-steroidal anti-inflammatory drugs (NSAIDs) are COX-competitive inhibitors used in cancer therapy and prevention [88]. Recent evidence suggests that impairment of T-cell activation and cytotoxic cellular response may play a role in BCG therapy failure [89]. Nogapendekin alfa-inbakicept (NAI), known as N-803, an IL-15 super agonist, is a fused protein from a human IL-15 variant bound to a dimeric human IL-15Ra domain/human IgG1 Fc. This IL-15-based immunostimulatory complex plays a crucial role in the activation and proliferation of natural killer (NK) cells as well as effector and memory T cells [90]. Hypothesizing that NAI may act as this secondary activation mechanism for NK and T cells that synergistically influences BCG efficacy, Chamie et al. studied three cohorts of BCG-unresponsive patients in an open-label, multi-institutional study [68]. Cohort A included patients with CIS with or without other NMIBC papillary disease treated with intravesical NAI plus BCG. Cohort C included patients treated with NAI alone. Finally, cohort B included patients with BCG-unresponsive HG Ta/T1 NMIBC who also received NAI plus BCG. The primary endpoint of the analysis was the incidence of CR at the 3- or 6-month assessment visit for cohorts A and C and the DFS rate at 12 months for cohort B. CR was achieved in 58 (71%) patients in cohort A. Specifically, the CR rates at 3, 6, and 12 months were 55%, 56%, and 45%, respectively. The median duration of CR was 26.6 months in responders. The cystectomy rate was 9% in responders. Among patients in cohort B, the DFS rate at 12 months was 55.4%, with a median DFS of 19.3 months. Of the patients who received NAI only (cohort C), CR was achieved at 3 months in only 2/10 (20%). For this reason, the recruitment of cohort C was closed following protocol-defined stopping rules. The safety profile was also evaluated: The most common treatment-emergent adverse events reported after administration of NAI plus BCG were mainly related to bladder instillation, with most of them graded 1 to 2 [68]. Thus, encouraging results were obtained with a CR rate of 71% for the BCG-unresponsive NMIBC CIS cohort treated with NAI plus BCG, which was higher than the 30% proposed by Kamat et al. as a target for clinical efficacy [91].

In the same setting, ALT-801 is a recombinant humanized T-cell receptor (TCR)-IL-2 fusion protein that enhances NK and T cells’ cytotoxic immune responses against p53-expressing tumor cells [86]. In a phase I trial (NCT01625260), Sonpavde et al. [92] preliminarily found that intravenous infusion of ALT-801 and Gemcitabine has promised durable clinical activity in BCG-resistant high-risk NMIBC patients defined as high-grade Ta, T1, or CIS, tumor size greater than 4 cm, or multifocal tumors. Patients who showed CR after the induction cycle received a maintenance cycle and underwent a response assessment. CR was observed in 3 patients and was durable (≥18 months) in 2 of them. Subgroup analyses have shown transient IFN-γ and IL-6 but not TNF-α and IL-10 induction after ALT-801 administration [92].

## 5. Gene Therapy

**Figure 4:**
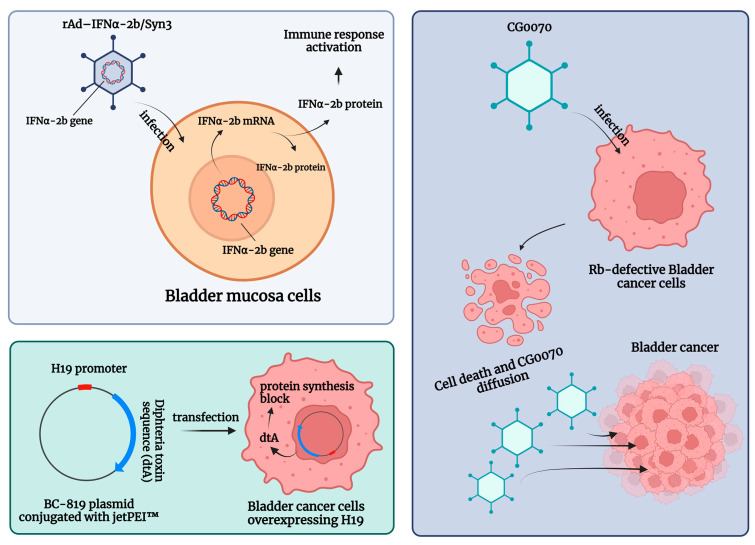
Schematic summary of the gene therapies for patients with non-muscle-invasive bladder carcinoma (NMIBC) unresponsive to Bacillus Calmette–Guerin (BCG) treatment. Abbreviations are as follows: dtA: diphtheria toxin; IFN: interferon; jetPEI™: polyethyleneimine; rAd-IFNα/Syn3: Nadofaragene firadenovec. The image was created with BioRender.com.

Genetic instability is an essential early step in the development of BC [93]. Such instability is easily detected at both the chromosomal and nucleotide levels. Consequently, microsatellite and chromosomal instability can be used as prognostic markers for BC, and previous experiences have shown that specific mutations are associated with a higher probability of progression and poorer survival after RC. In such a rapidly evolving tumor microenvironment (TME), genotypic and phenotypic alternations that can modulate gene and protein expression are critical for tumor invasion and the development of tumor escape mechanisms. Table 4 lists the studies addressing gene-delivery therapy for BCG-unresponsive NMIBC. A schematic summary of the gene therapies described is shown in Figure 4.

Intravesical recombinant IFNα-2b protein has been shown to be a well-tolerated molecule in BCG-unresponsive patients [101]. Here, intravesical IFNα gene delivery offers a new option for the local treatment of NMIBC by significantly prolonging the duration of exposure to IFNα-2b. Nadofaragene firadenovec (rAd-IFNα/Syn3) consists of rAdIFNα, a non-replicating recombinant adenovirus vector-based gene therapy agent that delivers a copy of the human IFNα-2b gene into the urothelial cell wall [102,103], while Syn3 is a polyamide surfactant that enhances viral transduction of the urothelium [104]. The in vitro reports showed that recombinant IFNα gene therapy led to local IFNα-2b production and was able to induce tumor regression [102,103]. In a phase II trial, in 40 HG NMIBC patients with BCG-refractory and/or relapsing disease, 35% of the included sample were free of HG recurrence after 12 months [95]. Following these encouraging results, Boorjian et al. evaluated 151 BCG-unresponsive patients within a phase III multicenter (33 US Institutions), single-arm, open-label, repeat-dose clinical study. The primary endpoint was any time CR in patients with CIS with or without an HG Ta/T1 NMIBC [94]. Overall, 55/103 (53.4%) CIS patients had a CR response within 3 months of the first dose, and this response was maintained in 25/55 (45.5%) of them at 12 months [94]. Nadofaragene firadenovec was well tolerated, with no dose-limiting toxicity or clinically significant treatment-related side effects, and a single dose was sufficient to achieve measurable urine IFNα [97]. Overall, rAd-IFNα/Syn3 has been evaluated in four single-armed cohort trials [94,95,97,98], which found CR rates ranging from 29% to 60% (3 months) and from 29% to 35% (12 months), respectively [6].

It is worth mentioning that in 2018, the FDA granted Fast Track and Breakthrough Therapy status to rAd-IFN/Syn3, and in 2022, this therapy called ALDASTRIN received Fast Track, Breakthrough Therapy, Accelerated Approval, and Priority Review status. Therefore, it is expected to be on the market soon.

CG0070 is a replication-competent oncolytic adenovirus that targets BC cells through their defective retinoblastoma (Rb) signaling pathway, expanding the field of emerging targeted agents. Packiam et al. studied 45 BCG-responsive patients who refused RC and received intravesical CG0070 in the context of a phase II single-arm multicenter trial (NCT02365818). Overall, administration of CG0070 resulted in a CR rate of 47% at 6 months for all patients and 50% for patients with pure CIS [96].

Another potential therapeutic option for BCG-unresponsive bladder cancers is BC-819, a double-stranded plasmid with an H19 promoter and a gene encoding for diphtheria toxin-A (dtA). This plasmid is conjugated with polyethyleneimine (in vivo-jetPEI™) to enhance cell transfection. The dtA synthesis is triggered by the transcription factors of H19, an oncofetal riboregulator RNA, which is overexpressed in embryonic tissues and various human tumors. Accordingly, the diphtheria toxin is selectively expressed in cancer cells, leading to blockage of protein synthesis and cell death. Sidi et al. tested the preliminary efficacy and safety of BC-189 in 18 BCG-unresponsive patients with confirmed expression of H19. In 22% (4 out of 18) of patients, tumor markers disappeared completely without the appearance of a new tumor. Monthly maintenance therapy was given to 9 patients, 5 of whom had a disease-free survival (DFS) greater than 35 weeks [100].

## 6. Other Therapies

**Figure 5:**
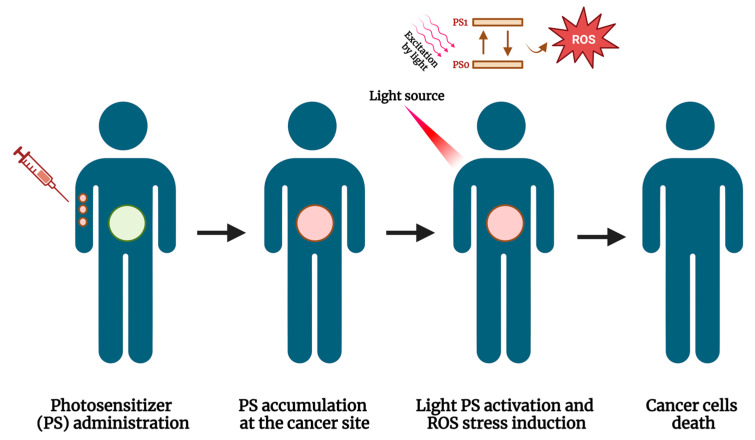
Working principle of photodynamic therapy (PDT). Abbreviations are as follows: PS0: ground state photosensitizer; PS1: excited photosensitizer. The image was created with BioRender.com.

Photodynamic therapy (PDT), which uses an interaction between absorbed light and a retained photosensitizing agent to destroy tissue (Figure 5), has been used to treat NMIBCs after BCG treatment [105]. Kulkarni et al. studied six BCG-unresponsive patients in an open-label, single-arm, dose-escalating study. The cohort was treated with photosensitizer TLD-1433-mediated PDT. Of three patients treated with the therapeutic dose, two achieved a CR after 180 days, which was confirmed at 18 months. The other patient was diagnosed with metastatic disease approximately 4 months later. PDT was well tolerated. However, all patients experienced at least one grade ≤ 2 adverse event [106]. Lee and colleagues evaluated the efficacy of PDT using Radachlorin in patients with HG NMIBC who were refractory or intolerant to BCG. By including 34 patients, the authors found an RFS rate of 90.9%, 64.4%, and 60.1% at 12, 24, and 30 months, respectively. No differences were found in survival analysis for lesion size, the presence of CIS, or the number of previous BCG cycles [107].

Targeting the vascular endothelial growth factor (VEGF) axis was studied in NMIBC patients. VEGF is a potent angiogenic factor and was first described as an essential growth factor for vascular endothelial cells, and sunitinib is a VEGF receptor (VEGFR) inhibitor with anti-tumor activity against BC. Zahoor et al. studied 19 NMIBC patients who had failed primary BCG administration in a single-arm phase II study (NCT01118351). Overall, 15/19 patients completed the full course of therapy. At 12-week cystoscopy, 44% of the study group showed remission, 50% had progressive disease, and 6 had BC recurrence. Thus, treatment with sunitinib was relatively safe but not associated with improved clinical outcomes in the target population [108].

Mutations in the fibroblast growth factor receptor 3 (FGFR3) gene have gained interest as a prognostic BC biomarker [20]. Hahn and the Hoosier Cancer Research Network Trial HCRN 12-157 evaluated the clinical and pharmacodynamic activity of dovitinib in a treatment-resistant, molecularly enriched non-muscle-invasive urothelial carcinoma of the bladder (NMIUC). Inclusion criteria were BCG-unresponsive NMIUC (>2 prior intravesical therapies) with increased phosphorylated FGFR3 (pFGFR3) expression evaluated centrally. The authors found an overall 6-month CR rate of 8% (33% among patients with FGFR3 mutation), whereas no response was observed in 11 mutation-negative FGFR3 patients. The reported DFS in the patient’s CR was 19 months [109].

Table 5 lists the reports considering alternative treatments for BCG-unresponsive NMIBC.

## 7. Conclusions and Future Perspectives

BCG-unresponsive disease constitutes a therapeutic challenge, and the best standard of care treatment continues to be investigated. Novel combinations that improve the efficacy of pembrolizumab are currently under investigation. ICIs T-cell immunoglobulin and ITIM domain (TIGIT) and lymphocyte-activation gene 3 (LAG-3) have been shown to contribute to treatment resistance in many malignancies, and their inhibition may enhance the effect of pembrolizumab [111]. In particular, TIGIT is an inhibitory receptor expressed on lymphocytes that has recently been highlighted as a major emerging target in cancer immunotherapy. By interacting with CD155 expressed on tumor cells, TIGIT can downregulate the NK functions of T cells. Thus, TIGIT has emerged as a key player in anti-tumor responses that can interfere with several steps of the cancer immunity cycle [112]. On the other hand, LAG-3 holds considerable potential as it suppresses T-cell activation and cytokine secretion, thus ensuring immune homeostasis. It shows remarkable synergy with PD-1 to inhibit immune responses. Immunotherapy targeting LAG-3 is moving forward in active clinical trials, also considering the scenario of BCG-unresponsive NIMBC, and the combination of anti-LAG-3 and anti-PD-1 has shown interesting efficacy in contrasting PD-1 resistance development [113]. The American Society of Clinical Oncology (ASCO) 2023 focuses on the KEYNOTE-057 (NCT02625961) cohort C, which will evaluate the efficacy and safety of the combination of pembrolizumab and vibostolimab (a TIGIT inhibitor) or favezelimab (a LAG-3 inhibitor) in patients with NIMBC. Patients will be randomly assigned in a 1:1 ratio. The primary efficacy endpoint will be the 12-month CR rate of HR NMIBC as determined by cystoscopy, cytology, biopsy, and radiologic imaging. A central pathology and radiology review will be applied [111].

In addition, encouraging results for erdafitinib, an oral selective pan-FGFR tyrosine kinase inhibitor, were recently highlighted by the THOR-2 study (NCT04172675) in patients with BCG-unresponsive CIS with FGFRalt with or without the papillary disease [114].

Tislelizumab (BGB-A317) is a humanized IgG4 anti-PD-1 monoclonal antibody designed to minimize binding to FcγR on macrophages. In preclinical studies, binding to FcγR on macrophages has been shown to compromise the anti-tumor activity of PD-1 antibodies through the activation of antibody-dependent macrophage-mediated killing of T effector cells. Previously, tislelizumab demonstrated clinical advantages in patients with locally advanced or metastatic PD-L1-positive UC [115]. Li et al. evaluated the efficacy and safety of tislelizumab plus radiotherapy as a bladder-sparing strategy in 14 BCG-unresponsive patients and found promising results with a bladder-preservation rate of 100% at 24 months and a remarkable OS rate at the same time point [116].

Enfortumab vedotin (EV) is an antibody–drug conjugate directed against Nectin-4. In EV-301, Powles et al. found an OS benefit for EV in patients with locally advanced or metastatic UC who had previously received platinum-based therapy and a PD-1 or PD-L1 inhibitor [117]. Kamat et al. presented EV-104 (NCT05014139) at ASCO 2023. This trial, a phase 1, open-label, multicenter, dose-escalation, and dose-expansion study, will evaluate the safety, tolerability, and anti-tumor activity of intravesical EV in BCG-unresponsive NMIBCs [118]. The study is currently enrolling in the US, with additional recruiting sites in Canada and Europe.

Many agents in single or combined administration have been evaluated in this scenario, obtaining disparate response rates. Starting from conventional chemotherapy up to immunotherapy and new targeted therapies, it seems plausible that the biological aggressiveness behind the BCG-unresponsive disease may harbor various vulnerabilities that can be targeted at different stages with different compounds, mirroring a remarkable interpatient and intratumor heterogenicity. As new therapeutic options emerge, selective biomarkers for treatment selection and clinical trial design are urgently needed. In addition, the optimal sequence of treatments remains to be elucidated. Therefore, novel therapeutics are currently being developed for patients with BC recurrence or persistence after BCG treatment. For example, Taizhou Hanzhong Biomedical Co., Ltd. (Taizhou, China) is supporting a single-arm, open-label, multicenter study to evaluate the efficacy and safety of HX008 in patients with BCG-unresponsive non-muscle-invasive bladder cancer [119] Similar to pembrolizumab, HX008 is a humanized anti-PD-1 monoclonal antibody that inhibits PD-1–PD-L1 binding, leading to T-cell activation [120]. This clinical trial started in September 2020, is in phase II, and is expected to be completed in December 2023.

Instead, enGene Inc. (Vancouver, BC, Canada) is sponsoring an open-label, multicenter study to evaluate the efficacy of intravesical administration of EG-70 in patients with BCG-unresponsive non-muscle-invasive bladder cancer [121]. EG-70 is a non-viral vector plasmid that encodes IL-12 and RIG1 to trigger, respectively, the adaptive and innate immune responses. The intravesical administration ensures a localized immune response by delivering the plasmids into the bladder mucosal tissue. The clinical trial was started in April 2021 and is expected to provide complete data in February 2026.

In conclusion, BCG continues to play a central role in the treatment of high-risk NMIBC. This immunotherapy is highly effective in preventing the recurrence and progression of NMIBC and remains the treatment of choice today. However, there is a significant proportion of patients who are classified as BCG-unresponsive. The aim of our review was to examine the role of these alternative therapies, focusing on the new molecular targets.

In addition to BCG, there are other intravesical therapies used in the treatment of NMIBC. Gemcitabine, docetaxel, valrubicin, and other therapies have shown a potential role in the treatment of urothelial carcinoma. All of these therapies aim to prevent tumor recurrence, treat high-risk or refractory NMIBC, or treat certain patients who cannot tolerate or do not respond to BCG. Our review highlights the role and limitations of these various drugs in the treatment of bladder cancer.

Hyperthermia has been studied as a potential treatment option for NMIBC. In combination with other therapies such as intravesical chemotherapy, hyperthermia has shown promise in improving treatment outcomes in NMIBC patients, particularly those with high-risk or recurrent tumors. Although hyperthermia has shown promising results in clinical trials, it is important to note that it is not yet considered the standard first-line treatment for NMIBC. Research is ongoing to determine the optimal protocols, safety, and long-term benefits of hyperthermia in combination with other treatments for NMIBC.

The use of immunotherapy and anti-inflammatory agents in NMIBC is an area of active research and ongoing clinical trials. Although some of these agents have shown promising results, further demonstration of their safety and efficacy in the treatment of NMIBC is needed. Advances in the field of immunotherapy hold great potential to transform the treatment of NMIBC and improve outcomes for patients with this type of bladder cancer. However, it is critical to recognize that not all patients will benefit from these therapies, and the selection of the most appropriate treatment should be guided by individual patient characteristics and clinical considerations.

Gene therapy for the treatment of NMIBC, while very promising, is still in the early stages of development, and further research is needed to optimize its efficacy and safety. Challenges in gene delivery, immune response regulation, and targeted gene expression need to be addressed. Clinical trials are currently underway to evaluate the safety and efficacy of gene therapy for NMIBC. Future advances in this field may provide new treatment options for patients with this type of bladder cancer.

## Figures and Tables

**Table 1:** Studies on intravesical administration of chemotherapy in NMIBC patients after BCG.

Author, Year	Patients Number	Study Design	Treatment	Regimen	Effects of Treatment	Main Findings
Chevuru et al., 2023 [25]	97	Retrospective	Gemcitabine Docetaxel	Induction +/− maintenance	Gemcitabine: inhibition of DNA synthesis Docetaxel: inhibition of cell division	12, 24, and 60 months RFS 57%, 44%, and 24% (12, 24, and 60 months HG-RFS 60%, 50%, and 30%, respectively); BCG unresponsive: 12, 24, and 60 months RFS 67%, 50%, and 28%; CIS-only: 1-, 2-, and 5-year RFS 48%, 38%, and 22%, papillary only: 1-, 2-, and 5-year RFS 64%, 49%, and 25% (*p* = 0.3); 12, 24, and 60 months RC-free survival 89%, 86%, and 75%
Hurle et al., 2020 [26]	36	Open-label, single-arm	Gemcitabine	Induction +/− maintenance	Gemcitabine: inhibition of DNA synthesis	12 and 24 months DFS 44.44% (95% CI 28.02–59.64%) and 31.66% (95% CI 16.97–47.43%); 12 and 24 months PFS 80.13% (95% CI: 62.78–90.00%) and 69.55% (95% CI: 50.33% 82.52%), 24 months CSS 80.68% (95% CI 61.49–90.96%), 24 months OS 77.9% (95% CI 58.78–88.92%)
Steinberg et al., 2020 [27]	276	Retrospective	Gemcitabine Docetaxel	Induction +/− maintenance	Gemcitabine: inhibition DNA synthesis Docetaxel: inhibition of cell division	1 and 2 years RFS 60% and 46% (43% if CIS present); HG 1 and 2 years RFS 65% and 52% (50% if CIS present)
DeCastro et al., 2020 [28]	18	Phase I trial	Cabazitaxel Gemcitabin Cisplatin	Induction +/− maintenance (cabazitaxel + gemcitabine)	Cabazitaxel: inhibition of cell division Gemcitabine: inhibition of DNA synthesis Cisplatin: inhibition of DNA replication and transcription	CR rate: 89%; PR: 94% (negative biopsy but positive cytology). One and two years RFS 0.83 (range 0.57 to 0.94) and 0.64 (0.32 to 0.84), median 27 months. One- and two-years RC-free survival 0.94 (0.67 to 0.99) and 0.81 (0.52 to 0.94)
Milbar et al., 2017 [29]	33 (22 BCG unresponsive/relapsing)	Retrospective	Gemcitabine Docetaxel	Induction +/− maintenance	Gemcitabine: inhibition DNA synthesis Docetaxel: inhibition of cell division	1 and 2 years DFS38% and 24%. One and two years HG-RFS 49% and 34%.
Dalbagni et al., 2017 [30]	19	Single-arm, phase I/II trial	Gemcitabine Everolimus (oral)	Gemcitabine induction + everolimus maintenance	Gemcitabine: inhibition of DNA synthesis Everolimus: mTOR inhibition	3, 6, and 9, 12 months RFS 58% (95% CI 33–76%), 27% (95% CI 9–49%) and 20% (95% CI 5–42%)
Robins et al., 2017 [31]	22	Single-arm, open-label, phase II trial	Nab-paclitaxel	Induction +/− maintenance	Nab-paclitaxel: inhibition of cell division	Overall CR 36%, non-CIS CR 63, CIS CR 25%. One and three-year RFS 32% and 18% (no CIS CR 40%, CIS CR 10%),
Cockerill et al., 2016 [32]	27	Retrospective	Gemcitabine MMC	Induction, no standardized maintenance	Gemcitabine: inhibition of DNA synthesis MMC: inhibition of DNA functions.	63% recurrence rate, median RFS 15.2 months (range 1.7–32). RFS 37% (median follow-up 22 months)
Steinberg et al., 2015 [33]	45	Retrospective	Gemcitabine Docetaxel	Induction +/− maintenance	Gemcitabine: inhibition of DNA synthesis Docetaxel: inhibition of cell division	Treatment success (no recurrence + no cystectomy) 66% at 12 weeks, 54% at 1 year, 34% at 2 years; median time to failure 3.1 months (range 2.2–25.9)
Lightfoot et al., 2014 [34]	52 (10 BCG naive)	Retrospective	Gemcitabine MMC	Induction +/− maintenance	Gemcitabine: inhibition of DNA synthesis MMC: inhibition of DNA functions.	CR 68%, 1- and 2-year RFS, 48%, and 38%
McKiernan et al., 2014 [35]	28	Single-arm, phase II trial	Nab-paclitaxel	Induction +/− maintenance	Nab-paclitaxel: inhibition of cell division	35.7% CR, 1 and 2-year RFS 35.7% and RFS 30.6%. 12, 24, and 36 months CFS 74%, 74%, and 55%
Barlow et al., 2013 [36]	54	Retrospective	Docetaxel	Induction +/− maintenance	Docetaxel: inhibition of cell division	59% CR (cystoscopy with biopsy + cytology); 1- and 3-year RFS 40% and 25%; 31% RC rate
Skinner et al., 2013 [37]	58	Single-arm, open-label, phase II trial	Gemcitabine	Induction +/− maintenance	Gemcitabine: inhibition of DNA synthesis	3 months CR (negative cystoscopy, urinary cytology +/− biopsy) 47%. Median RFS 6.1 months (95% CI 16–43), 21% at 24 months. Progression/RC rate 36%.
Sternberg et al., 2013 [38]	69	Retrospective	Gemcitabine	Induction	Gemcitabine: inhibition of DNA synthesis	5 years progression rate 19% for BCG-refractory pts, 22% for pts with other types of BCG failure (HR 1.09, 95% CI 0.34–3.50). CR (negative cystoscopy and cytology) in 27 pts, PR (negative cystoscopy and positive cytology in 19 with), NR (positive cystoscopy) 20 pts. Subsequent RC in 20 pts
Steinberg et al., 2011 [39]	90	Single-arm, pivotal phase III open-label study	Valrubicin	Induction	Valrubicin: inhibition of DNA and RNA synthesis	CR in 18 pts at 3 and 6 months (negative cytology, cystoscopy, and biopsy), NR 64 pts
McKiernan et al., 2011 [40]	18	Phase I trial	Nab-paclitaxel	Induction	Nab-paclitaxel: inhibition of cell division	CR in 5 patients (28%), 13 NR (stage progression in 1)
Bassi et al., 2011 [41]	16	Single-arm, open-label, phase I trial	Paclitaxel-hyaluronic acid	Induction	Paclitaxel: inhibition of cell division	6 NR (40%), 9 disease-free pts (60%)
Di Lorenzo et al., 2010 [42]	80	Multicentric, phase II trial, randomized	Gemcitabine vs. BCG	Induction + maintenance for both arms	Gemcitabine: inhibition of DNA synthesis BCG: stimulating cellular and humoral immune response	2-year RFS 19% for Gem (95% CI, 5–39), 3% for BCG (95% CI, 0–21; HR, 0.15; 95% CI, 0.1–0.3.008). Progression rate 33% for Gem, 37.5% for BCG
Perdonà et al., 2010 [43]	20	Single-arm, phase II trial	Gemcitabine	Induction + maintenance	Gemcitabine: inhibition of DNA synthesis	3 months CR at the first 75%; 55% recurrence rate (11 of 20 pts); 45% progression rate (5 of 11 pts)
Laudano et al., 2010 [44]	18	Single-arm, phase I trial	Docetaxel	Induction	Docetaxel: inhibition of cell division	22% CR, 17% PR (NMIBC recurrence requiring TURBT with no further treatment), 61% NR (RC or further pharmacologic therapy). PFS 89%.
Addeo et al., 2010 [45]	109	Phase III trial randomized	Gemcitabine vs. MMC	Induction +/− maintenance for both arms	Gemcitabine: inhibition of DNA synthesis MMC: inhibition of DNA functions.	RFS in gemcitabine arm, 72% (39 of 54 pts), in MMC arm 61% (33 of 55 pts). Stage progression in 10 pts in the MMC arm and 6 in the gem arm
Ignatoff et al., 2009 [46]	38	Multicentric, single-arm, phase II trial	AD32 (doxorubicin analog with limited systemic exposure)	Induction	AD32: inhibition of DNA functions and induction of apoptosis	CR 42.9% (90% CI: 24.5%, 62.8%), CIS CR 23.8% (90% CI: 9.9%, 43.7%). 12 and 24 RFS months 20% (90% CI: 7.8–36.1%) and 15% (CI, 4.9%, 30.2%),12 and 24 CIS RFS 80% (90% CI, 31.4%, 95.8%) if previous CR. PFS 22.4 months, CIS PFS 8.7 months
Mohanty et al., 2008 [47]	35	Single-arm, non-randomized, phase I trial	Gemcitabine	Induction	Gemcitabine: inhibition of DNA synthesis	At 18 months follow-up 21 disease free pts (60%), 11 pts (31.4%) with superficial recurrences, 3 (8.75%) with MIBC. Average RFS 12 months, average time to progression 16 months.
Gunelli et al., 2007 [48]	40	Single-arm, phase II trial	Gemcitabine	Induction	Gemcitabine: inhibition of DNA synthesis	95% (38 of 40 pts) CR at 6 months (cystoscopy + cytology); overall event-free survival rate 80% at 1 year and 66% at 2.5 years. At a median follow-up of 28 months, 35% relapse rate (NMIBC). RC in 2 pts
Dalbagni et al., 2006 [49]	30	Single-arm, phase II trial	Gemcitabine	Induction	Gemcitabine: inhibition of DNA synthesis	50% CR; median RFS 3.6 months (95% CI, 2.9 to 11.0 months); 21% 1-year RFS in pevious CR (95% CI, 0% to 43%). RC rate 37%
McKiernan et al., 2006 [50]	18	Single-arm, phase I trial	Docetaxel	Induction	Docetaxel: inhibition of cell division	CR in 56% (10 pts)
Bartoletti et al., 2005 [51]	40 BCG refractory (total population 116)	Multicentric, single-arm, phase II trial	Gemcitabine	Induction	Gemcitabine: inhibition of DNA synthesis	Recurrence rate 32.5%, relapse in 6 (25%) of 24 intermediate-risk BCG refractory pts and 7 (43.7%) of 16 BCG refractory high-risk pts
Bassi et al., 2005 [52]	9	Single-arm, phase I trial	Gemcitabine	Induction +/− maintenance	Gemcitabine: inhibition of DNA synthesis	CR in 4/9 pts
Dalbagni et al., 2002 [53]	14	Single-arm, phase I trial	Gemcitabine	Induction (dose levels 500 mg, 1.000 mg, 1.500 mg, and 2.000 mg.	Gemcitabine: inhibition DNA synthesis	CR (defined as a negative posttreatment cystoscopy with biopsy of the urothelium + negative cytology) in 7, failure in 11 (negative bladder biopsy + persistent positive cytology), RC rate 1/11 pts

Abbreviations are as follows: BCG: Bacillus Calmette–Guerin; CFS: cystectomy-free survival CIS: carcinoma in situ; CR: complete response; CRR: complete response rate; CSM: cancer-specific mortality; CSS: cancer-specific survival; Gem/Doce: gemcitabine/docetaxel; HG: high grade; IFN: interferon; MIBC: muscle-invasive bladder cancer; MMC: mitomycin C; Nab: nanoparticle albumin-bound; NMIBC: non-muscle-invasive bladder cancer; NR: non-responders; OS: overall survival; PFS: progression-free survival; PR: partial response; Pts: patients; RC: radical cystectomy; RFS: recurrence-free survival; TNF: tumor necrosis factor; TRAIL: tumor necrosis factor-related apoptosis-inducing ligand.

**Table 2:** Chemo-hyperthermia and MMC administration in NMIBC patients following BCG.

Author, Year	Patients Number	Study Design	Treatment	Regimen	Effects of Treatment	Main Findings
Marquette et al., 2020 [59]	22	Retrospective	HIVEC MMC	Induction	MMC: inhibition of DNA functions. Hyperthermia: DNA damage, ROS generation, cell death induction, damage to tumor vascular system, activation of immune response.	Progression to MIBC: 2 pts (9.1%) Time to to RC: 4 pts (18.2%)
De Jong et al., 2018 [60]	55	Post hoc analysis of prospective data	MMC + hyperthermia	Induction + maintenance	MMC: inhibition of DNA functions. Hyperthermia: DNA damage, ROS generation, cell death induction, damage to tumor vascular system, activation of immune response.	Median DFS 17.7 months, papillary only DFS 28.8 months, papillary + CIS DFS 17.7 months, T1/ T1 + CIS DFS 12.1 months CR 70% at 3 months if concomitant CIS. One-year disease recurrence/progression 53%. Overall CR 50%, recurrence rate 42% and progression rate 8%. RC rate 50%
Tan et al., 2019 [61]	104 (48 randomized to MMC + hyperthermia)	Phase III trial, open-label, randomized with control	MMC + hyperthermia vs. BCG or institutional standard of care defined at randomization	Induction +/− maintenance	MMC: Inhibition of DNA functions. Hyperthermia: DNA damage, ROS generation, cell death induction, damage to tumor vascular system, activation of immune response. BCG: stimulating cellular and humoral immune response.	No statistically significant differences between the 2 arms in 24 months DFS (35% vs. 41%, HR 1.33, 95% CI 0.84–2.10, *p* = 0.23; adjusted *p* = 0.49) and 3 months CIS CR (30% vs. 47%, OR 0.43, 95% CI 0.18–1.28, *p* = 0.15). Lower DFS of experimental arm lower if CIS (HR 2.06, 95% CI 1.17–3.62, *p* = 0.01). 24 months DFS in non-CIS pts for experimental and control arms 53% and 24%,. worse DFS in papillary tumor + CIS than in CIS only. No differential treatment effect in CIS only pts (HR 1.53, 95% CI 0.77–3.05, *p* = 0.22). No difference between the arms in PFS (24 month rates 83% vs. 87%, *p* = 0.16), and RFS (24 month rates 23% vs. 40%; *p* = 0.98), borderline difference in DSS (24 month rates 89% vs. 96%; *p* = 0.04). Progression in 4 pts in both arms.
Soria et al., 2016 [62]	34	Multicentric, single-arm, phase I–II trial	MMC + hyperthermia	Induction	MMC: inhibition of DNA functions. Hyperthermia: DNA damage, ROS generation, cell death induction, damage to tumor vascular system, activation of immune response.	Response rate 59%, 42 NR, 1-year progression rate 18%; 41 months DFS 44.1%. RFS 10.5 months, PFS 29.5 months, RC-free survival 20 months (range 8–60). At median follow-up of 41 months, recurrence and progression rates 35.3 and 23.5%
Inman et al., 2014 [63]	15	Pilot prospective trial	MMC + hyperthermia	Induction +/− maintenance	MMC: inhibition of DNA functions. Hyperthermia: DNA damage, ROS generation, cell death induction, damage to tumor vascular system, activation of immune response.	67% recurrence rate, RFS 15.4 months, 60% RC rate, RC-free survival 20.1 months
Witjes et al., 2009 [64]	51 (17 BCG refractory, 15 BCG relapsing)	Retrospective	MMC + hyperthermia	Induction + maintenance	MMC: inhibition of DNA functions. Hyperthermia: DNA damage, ROS generation, cell death induction, damage to tumor vascular system, activation of immune response.	CR (negative cytology and biopsy) 92%, 50% at 2 years. 49% recurrence rate, mean RFS 27 months (median 22 months).
Nativ et al., 2009 [65]	111	Retrospective	MMC + hyperthermia	Induction + maintenance	MMC: inhibition of DNA functions. Hyperthermia: DNA damage, ROS generation, cell death induction, damage to tumor vascular system, activation of immune response.	1 and 2 year DFS 85% and 56%, respectively; average DFS 16 months; 3% progression rate to MIBC. Two-year recurrence rate of 61% if maintenance not administered, 39% if administered.

Abbreviations: CI: confidence interval; CIS: carcinoma in situ; CR: complete response; HIVEC: hyperthermic intravesical chemotherapy; MIBC: muscle-invasive bladder cancer; MMC: mitomycin C; NK: natural killer; RC: radical cystectomy; RFS: recurrence-free survival; ROS: reactive oxygen species; PFS: progression-free survival; Pts: patients.

**Table 3:** Immunotherapy and inflammation-targeted agents’ administration in NMIBC patients following BCG.

Author, Year	Patients Number	Study Design	Treatment	Regimen	Effects of Treatment	Main Findings
Meghani et al., 2022 [69]	9	Single-arm, phase I trial	BCG + pembrolizumab (intravesical)	Induction + maintenance (pembrolizumab only)	BCG: stimulating cellular and humoral immune response. Pembrolizumab: stimulation of immune response.	3, 6, and 12 months RFS 100%, 67% (95% CI: 42–100%) and 22% (95% CI: 7–75%), respectively. Median RFS 6.2 months. Progression in 5 pts, 6 and 12 months PFS 100% and 56% (95% CI: 31–100%)
Chamie et al., 2022 [68]	171 (cohorts A and C: pts with BCG-unresponsive CIS with or without Ta/T1 papillary NMIBC; cohort B pts with BCG-unresponsive high-grade Ta/T1 papillary NMIBC)	Pivotal trial, multicentric open-label, single-arm, three- cohort	IL-15 superagonist NAI (intravesical) + BCG vs. NAI (intravesical) alone	Induction +/− maintenance	NAI: stimulation of immune response. BCG: stimulating cellular and humoral immune response.	Cohort A: 71% 3, 6, and 12 months CR 55% (45 of 82 patients; 95% CI = 43.5% to 65.9%), 56% (46 of 82 patients; 95% CI = 44.7% to 67.0%), and 45% (37 of 82 patients; 95% CI = 34.1% to 56.5%). 24 months PFS 84.7%%. 7% RC rate in CR pts, 33% in NR Cohort B: 12, 18 and 24 months DFS 55.4% (95% CI = 42.0% to 66.8%), 51.1% (95% CI = 37.6% to 63.1%) and 48.3% (95% CI = 34.5% to 60.7%). RC rate 7%. Cohort C: 3 months CR 20%, 10% of pts with CR maintained at 6 months
Alanee et al., 2021 [67]	18	Single-arm, phase I trial	BCG + intravenous pembrolizumab	BCG induction + 6 doses of pembrolizumab every 3 weeks concurrently with BCG	BCG: stimulating cellular and humoral immune response Pembrolizumab: stimulation of immune response.	CR 69% at 3 months following BCG treatment. One patient progressed to MIBC.
Balar et al., 2021 [10]	96	Open-label, single-arm, multicentric, phase II trial	Pembrolizumab (intravenous)	Every 3 weeks up to 24 months	Pembrolizumab: stimulation of immune response.	39 pts (41%; 95% CI 30∙7–51∙1) achieved CR at 3 months, median duration of CR 16.2 months (95% CI 6∙7–36∙2), 51% recurrence after CR. At 12 months PFS to worsening of grade or stage or death 83% (95% CI 70.22–90.4), PFS to MIBC or metastatic disease or death 97% (86.0–99.2).
Li et al., 2017 [70]	94	Retrospective	MCNA	Induction +/− maintenance	MCNA: stimulation of immune response, apoptosis induction.	6, 12, and 24 months DFS 48.9% (95% CI 38.0–59.0%), 34.8% (95% CI 24.7–45%) and 28.3% (15.7–34.3%). Papillary-only 6, 12, and 24 months DFS 61.2% (95% CI 38.2–77.8%), 61.2% (95% CI 38.2–77.8%) and 50.1% (95% CI 27.5–69.0%) at 2 years. In pts with CIS +/− papillary disease 6, 12 and 24 months DFS 44.8% (95% CI 32.3–56.4%), 26.5% (95% CI 16.3–37.9%) and 16.6% (95% CI 8.6–26.9%). 47.9% RC rate, 16% progression rate to MIBC and 11.7% to metastatic disease.
Huang et al., 2017 [71]	1	Case report	ALT-803 (NAI) + BCG	Induction	ALT-803 (NAI): stimulating immune response. BCG: stimulating cellular and humoral immune response.	No evidence of recurrence at 19 months after treatment.
Morales et al., 2015 [72]	129	Open-label multicentric, single-arm phase II trial	MCNA	Induction +/− maintenance	MCNA: stimulation of immune response, apoptosis induction.	6, 12, and 24 months CR 34.1%, 22.5% and 14.7% at (34%, 195%, and 11% in CIS pts). 21.7% progression rate, 6, 12, 24, and 36 months PFS 95%, 87.3%, 79.8%, and 77.7%. RC rate 43%.
Kowalski et al., 2012 [73]	46	Open-label, multicentric, 2-arm phase I trial	Oportuzumab monatox	Induction +/− maintenance	Oportuzumab monatox: apoptosis induction	CR 44%, 16% at 1 year. One-year recurrence rate 65%. Two pts progressed to MIBC.
Morales et al., 2009 [74]	55	Single-arm, phase II trial	MCNA	Induction +/− maintenance	MCNA: stimulation of immune response, apoptosis induction.	CR 27.3% at 12 and 26 weeks, 31.8% and 22.7% at 12 and 18 months in the group receiving 4 mg, 46.4% at 12 and 26 weeks, 25% and 28.6% at 12 and 18 months in the group receiving 8 mg.
Joudi et al., 2006 [75]	1106 (467 BCG failure)	Multicentric, single-arm, phase II trial	BCG + IFNα2b (intravesical)	Induction +/− maintenance	BCG: stimulating cellular and humoral immune response. Interferon alpha-2b: induction of cytotoxic effects	24 months disease-free rate 45%
O’Donnel et al., 2004 [76]	490 (231 BCG failure)	Phase II trial	BCG + IFNα2b (intravesical)	Induction + maintenance	BCG: stimulating cellular and humoral immune response. Interferon alpha 2 b: induction of cytotoxic effects	In BCG failure group, recurrence rate 51.5%, 20.8% at 3 months. 24 months disease-free rate 42%, median RFS 16 months. Progression rate to MIBC 4.3%, to metastatic disease 2.6%. RC rate 3.9%
Lam et al., 2003 [77]	32 (20 BCG failure)	Retrospective	BCG + IFNα2b (intravesical)	Induction +/− maintenance	BCG: stimulating cellular and humoral immune response Interferon alpha 2 b: induction of cytotoxic effects.	In BCG failure group, 60% disease-free rate, 40% 3-month failure rate. Three pts underwent RC.
O’Donnell et al., 2001 [78]	40	Multicentric phase II trial	BCG + IFNα2b (intravesical)	Induction + maintenance	BCG: stimulating cellular and humoral immune response Interferon alpha-2b: induction of cytotoxic effects	12, 24, and 30 months RFS 63%, 53%, and 55%
Sarosdy et al., 1998 [79]	86 (60 BCG-resistant CIS, 26 with BCG-intolerant CIS)	Single-arm, phase II trial	Bropirimine	Oral self-administration, 3.0 g/day for 3 consecutive days, weekly, up to 1 year	Bropirimine: stimulation of immune response	CR in 14 pts (30%, 95% CI 16.7 to 42.9) of 47 evaluable BCG-resistant pts, 2 additional pts free of CIS (negative biopsy and negative cytology) but recurrent papillary tumor. Overall CR in 21 pts (24%). RFS in CR pts 65–810 days. RC in 26 pts. Progression to MIBC or metastatic disease in 4 pts (6%).

Abbreviations: CI: confidence interval; CR: complete response; DFS: disease-free survival; DSS: disease-specific survival; CIS: carcinoma in situ; EpCAM: epithelial cell adhesion molecule; IFN: interferon; IL: interleukin; MCNA: mycobacterium phlei cell wall-nucleic acid complex; MIBC: muscle-invasive bladder cancer; NAI: nogapendekin alfa inbakicept; PD-1: programmed cell death protein 1; RC: radical cystectomy; RFS: recurrence-free survival; TNF: tumor necrosis factor.

**Table 4:** Gene-delivered agents’ administration in NMIBC patients following BCG.

Author, Year	Patients Number	Study Design	Treatment	Regimen	Effects of Treatment	Main Findings
Boorjian et al., 2021 [94]	151	Multicentric open-label repeat-dose phase III trial	rAd-IFNa/Syn3 (intravesical)	Induction +/− maintenance	rAd–IFNa2b/Syn3: cytotoxic effects	3-month CR: 55 (53.4%) CIS pts (with or without a high-grade Ta or T1 tumor). 12-month maintained CR among 25/55 (45.5%).
Shore et al., 2017 [95]	40	Phase II trial, randomized,	rAd–IFNα-2b/Syn3 (intravesical)	Induction +/− maintenance	rAd–IFNa2b/Syn3: cytotoxic effects	12-month RFS: 35% Time to HG recurrence: 6.5 months. RC rate: 35%. 11 pts were disease-free for 15 to more than 36 months.
Packiam et al., 2018 [96]	45	Open-label, multicentric, single-arm, interventional phase II trial	CG0070 (replication selective serotype-5 oncolytic adenovirus)	Induction +/− maintenance	CG0070: stimulation of immune response for Rb-defective tumor cells	Overall CR: 47% (95% CI: 32–62) CR in CIS: 58% (95% CI: 37–78) CR in CIS ± Ta/T1: 50% (95% CI: 33–67) CR CIS + Ta/T1: 33% (95% CI: 10–65) CR in pure Ta/T1: 33% (95% CI: 8–70) 4 pts developed MIBC over 12-month follow-up 3 cancer-specific deaths
Navai et al., 2016 [97]	7	Single-arm, phase Ib trial	rAd–IFNα-2b/Syn3 (intravesical)	Two administrations +/− second treatment	rAd–IFNa2b/Syn3: cytotoxic effects in selected bladder mucosa cells.	CR in 2 pts (29%). Thereafter, RC in 6 pts (1 was lost to follow-up), and 1 pts died of the disease.
Dinney et al., 2013 [98]	17	Open-label, dose-escalating, non-randomized, multicentric phase I trial	rAd–IFNα-2b/Syn3 (intravesical)	Single treatment (3 patients received a second dose)	rAd–IFNa2b/Syn3: cytotoxic effects in selected bladder mucosa cells.	CR in 7 patients at 3 months, RFS up to 39.2 months. RC performed in 10 pts.
Burke et al., 2012 [99]	35	Single-arm, observational, phase I trial	CG0070 (replication selective serotype-5 oncolytic adenovirus)	Induction +/− maintenance	CG0070: stimulation of immune response for Rb-defective tumor cells	Overall RR: 48.6% CR duration: 10.4 months in responders (with some responses ongoing at 17.0 months) CR 50.0% in pts with CIS only CR in pts with CIS alone or CIS + Ta or T1 tumors: 41.2% Higher CR (58.3%) in Rp-positive pts
Sidi et al., 2008 [100]	18	Single-arm, phase I/II trial	BC-819 DNA plasmid (intravesical)	Induction +/− maintenance	BC-8129 DNA plasmid: inhibition of protein synthesis and induction of cell death.	Overall CR: 22% 35 weeks DFS: 4 pts 49 weeks DFS: 1 pt

Abbreviations are as follows: CIS: carcinoma in situ; CR: complete response; DFS: disease-free survival; GM-CSF: granulocyte macrophage-colony stimulating factor; MIBC: muscle-invasive bladder cancer; Rb: retinoblastoma; RFS: recurrence-free survival; RR: response rate; RC: radical cystectomy.

**Table 5:** Other agents’ administration in NMIBC patients following BCG.

Author, Year	Patients Number	Study Design	Treatment	Regimen	Effects of Treatment	Main Findings
Kulkarni et al., 2022 [106]	6	Single-arm, phase Ib trial	PDT: green light laser (wavelength 520 nm) + intravesical TLD–1433 (ruthenium-based photosensitizer)	Single treatment	Oxygen-reactive species production: cell death induction.	Disease persistence in 3 pts treated with 0.35 mg/cm2 TLD–1433 dose, CR at 3 and 6 months in 2 pts treated with 0.70 mg/cm^2^ (DFS 18 months), metastatic progression in 1 case
Zahoor et al., 2018 [108]	19	Single-arm, phase II trial	Sunitinib	12 weeks therapy	Multi-targeted receptor tyrosine kinase (RTK) inhibition: inhibition of cell growth.	DFS 44% at 12 weeks, 50% progression rate, one recurrence. At 12 months 4 pts remained disease free, overall 78% progression rate, median time to progression 4.7 months
Hahn et al., 2017 [109]	13	Single-arm, phase II trial	Dovitinib	4 weeks therapy cycles (median 4 cycles, range 1–19)	Multi-targeted receptor tyrosine kinase (RTK) inhibition: inhibition of cell growth.	CR in 1 case out of 3 pts with FGFR3 mutation (33%), no response in 11 pts (85%), and progression to MIBC in 1 case (8%). DFS in the CR patient 19 months. RC rate 62% (11 pts)
Lee et al., 2013 [107]	34 (BCG refractory + intolerant)	Retrospective	PDT: semiconductor laser (power 3 W, wavelength 662 ± 2 nm) + intravenous Radachlorin	Single treatment	Caspase-3 activation and induction of cell death.	12, 24, and 30 months DFS 90.9%, 64.4%, and 60.1%, respectively. RC performed in 2 pts
Berger et al., 2003 [110]	31 (10 with previous BCG treatment)	Retrospective	PDT: laser (maximal power 7 W, wavelength of 633 nm—red light) + intravesical 5ALA	1 to 6 treatment sessions	Mitochondrial cytochrome C translocation and induction of cell apoptosis.	40% RFS at 11.8 months (range 1–26), 60% recurrence rate after a mean follow-up of 12.7 months. Three patients receiving repeated treatment (3 sessions) were disease free at 6.3 months (range 1–11)

Abbreviations are as follows: 5ALA: 5-aminolevulinic acid; BCG: Bacillus Calmette–Guérin; CR: complete response; FGFR: fibroblast growth factor receptor; PDT: photodynamic therapy; MIBC: muscle-invasive bladder cancer; RC: radical cystectomy; RFS: recurrence-free survival; Pts: patients; VEGF: vascular endothelial growth factor.

## Data Availability

Data sharing not applicable.
